# Long Non-Coding RNAs in Kidney Disease

**DOI:** 10.3390/ijms20133276

**Published:** 2019-07-03

**Authors:** Michael Ignarski, Rashidul Islam, Roman-Ulrich Müller

**Affiliations:** 1 Department II of Internal Medicine and Center for Molecular Medicine, University of Cologne, Faculty of Medicine and University Hospital Cologne, 50931 Cologne, Germany; 2 Cologne Excellence Cluster on Cellular Stress Responses in Aging-Associated Diseases, University of Cologne, Faculty of Medicine and University Hospital Cologne, 50931 Cologne, Germany; 3 Systems Biology of Ageing Cologne, University of Cologne, 50931 Cologne, Germany

**Keywords:** lncRNA, long non-coding RNA, miRNA, kidney, glomerulus, podocyte, acute kidney injury, AKI, diabetic nephropathy

## Abstract

Non-coding RNA species contribute more than 90% of all transcripts and have gained increasing attention in the last decade. One of the most recent members of this group are long non-coding RNAs (lncRNAs) which are characterized by a length of more than 200 nucleotides and a lack of coding potential. However, in contrast to this simple definition, lncRNAs are heterogenous regarding their molecular function—including the modulation of small RNA and protein function, guidance of epigenetic modifications and a role as enhancer RNAs. Furthermore, they show a highly tissue-specific expression pattern. These aspects already point towards an important role in cellular biology and imply lncRNAs as players in development, health and disease. This view has been confirmed by numerous publications from different fields in the last years and has raised the question as to whether lncRNAs may be future therapeutic targets in human disease. Here, we provide a concise overview of the current knowledge on lncRNAs in both glomerular and tubulointerstitial kidney disease.

## 1. Introduction

Most of the human genome is actively transcribed but less than 2% contains protein coding transcripts (mRNA). The other transcripts produced show no or low coding potential and have, therefore, been summarized in the large group of non-coding RNAs (ncRNA). This group contains the long known ribosomal RNAs (rRNAs) and transfer RNAs (tRNAs) involved in protein synthesis as well as two very diverse classes of ncRNAs mainly divided by their length: the class of small non-coding RNAs containing transcripts with lengths of less than 200 nucleotides consists of small interfering RNAs (siRNAs), small nuclear RNAs (snRNA), small nucleolar RNAs (snoRNAs), PIWI-interacting RNAs (piRNAs) and microRNAs (miRNAs). The role of miRNAs in kidney disease has been investigated extensively and reviewed multiple times over the last decades [1,2], therefore, we focus on the class of long non-coding RNAs (lncRNAs) with lengths of over 200 nucleotides. Like mRNAs most lncRNAs have their own promoters, are RNA polymerase II transcribed, 5’-capped, polyadenylated and subjected to splicing [3]. LncRNA genes are dispersed throughout the genome, they can be inter- or intragenic, in the latter case positioned in sense or antisense direction, inside exons, introns or overlapping both. Intergenic lncRNAs can be located at great distance from proteins coding genes, in close proximity or divergently transcribed from protein coding gene promoters. They can also be expressed from silencer, enhancer and insulator loci. For details on genomic location of lncRNAs refer to Laurent et al. [4]. So far, there is no formal classification with respect to lncRNA localization or function. Many lncRNAs were shown to act as nucleo- or cytoplasmic scaffolds providing platforms for interactions between other cellular components (e.g., DNA, proteins and other RNA species), which implies lncRNAs as central players in epigenetic processes and chromatin regulation. Another superordinate function of lncRNAs is the competition for binding sites, this can be the competitive binding to open chromatin leading to displacement of transcription factors or the sequestration of miRNAs leading to reduced inhibition of the target mRNA. Likewise, lncRNAs can act similarly to miRNAs to enhance or decrease the stability of mRNA. The great variety of lncRNA cellular functions was recently reviewed by Yao et al. [5].

Due to their generally low expression levels the vast majority of the 215,008 annotated human lncRNAs (RNAcentral, 5 May 2019) was only discovered within the last decade [6]. Whilst the existence of lncRNAs as a biotype is well conserved in mammals, identifying the actual homologues between species is more challenging than for protein-coding transcripts. LncRNAs are often not well-conserved on the sequence level but rather regarding structure and/or genomic position [7]. The expression pattern of lncRNA genes was shown to be far more tissue- and cell-type specific than is the case for protein coding genes [8,9]. Quantitative studies based mainly on RNA sequencing have led to a rapid growth of this field and have shown dysregulation of lncRNAs in many diseases [10]. Whilst before 2010 only few studies on this topic were published each year, this changed tremendously in the last decade and several thousand publications on lncRNAs are found for the year 2018. As to lncRNAs in the kidney, this rise in publication numbers occurred around five years later and resulted in about 100 studies in the year 2018. However, especially in the beginning much of this work was merely descriptive and did not focus on kidney disease but rather renal cell carcinoma due to the ease of analyses regarding RNA expression changes in tumors. Taking this into consideration, the aim of this review is filling the gap towards non-tumorous kidney diseases to lay a foundation for future studies building on existing data. 

## 2. LncRNAs in Glomerular Disease

**Figure 1:**
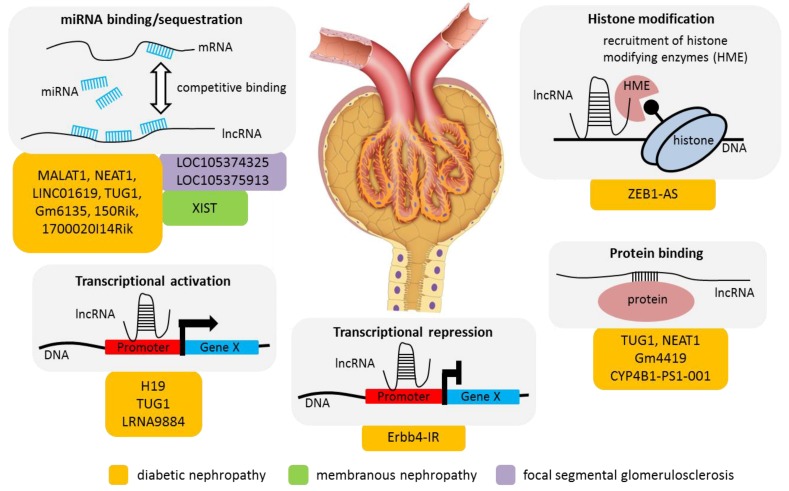
Long non-coding RNAs (lncRNAs) in glomerular disease and their reported molecular functions. Grey colored boxes depict the molecular mechanisms of lncRNA function (enhancer RNA, transcriptional repression, transcription activation, protein binding, miRNA binding/sequestration and histone modification) reported to play a role in glomerular disease. Orange, green and purple colored boxes indicate the disease: diabetic nephropathy, membranous nephropathy and focal segmental glomerulosclerosis, respectively. The lncRNAs associated with the particular mechanism and disease are specified by name. Glomerulus image obtained from: Aldona Griskieviciene/shutterstock.com.

As to glomerular disease by far most publications have analyzed the role of lncRNAs in diabetic nephropathy (Figure 1). Consequently, this review contains a focused paragraph on diabetic nephropathy but also provides an insight into the publications on other glomerular disease entities such as focal segmental glomerulosclerosis and membranous nephropathy (Table 1).

### 2.1. Diabetic Nephropathy—The Link between MicroRNAs and LncRNAs

With the incidence of diabetes mellitus rising and diabetic nephropathy (DN) being a leading cause of end-stage renal disease in Western societies [11,12], it is clear that researchers chose this entity as a key topic to elucidate the role of lncRNAs in kidney disease. Most of the work that goes beyond a mere description of non-coding RNA expression in DN focused on miRNAs and a few annotated lncRNAs the function of which had been addressed in other diseases/organs before. Despite the fact that miRNAs are not the topic of this review, it is important to note the intricate connection between miRNAs and lncRNAs. Firstly, the latter can impact miRNA function, e.g., by serving as miRNA sponges that inhibit their binding to the actual mRNA targets [13]. This has been described in the context of DN for a number of miRNA—lncRNA interactions including work on the lncRNAs TUG1, NEAT1 and MALAT1 [14,15,16]. Furthermore, lncRNA genes can harbor miRNAs that are set free by posttranscriptional cleavage. Prominent examples are the lncRNA PVT1 serving as a host of miRNA 1207-5p and both non-coding RNAs having been implicated in DN [17]. Importantly, miRNAs are often organized as clusters with four more miRNAs having been localized to the *PVT1* locus [18] all of which are upregulated by high glucose levels and impact extracellular matrix (ECM) formation. MiRNA clusters contained in lncRNAs can get very large as demonstrated by a megacluster of more than 40 miRNAs harbored in lnc-MGC. This cluster is induced in the glomeruli of several mouse models of diabetic nephropathy through endoplasmic reticulum (ER) stress signaling and responds to both high glucose and TGFβ-activation [19]. Inhibition of lnc-MGC using a “Gapmers”—antisense oligonucleotides that induce the RNaseH-mediated degradation of their targets often used in the lncRNA field—ameliorates several histological signs of diabetic nephropathy in a mouse model pointing towards a therapeutic potential of these findings.

### 2.2. Diabetic Nephropathy—Tthe Role of Specific Long Non-Coding RNAs (LncRNAs)

Regarding the involvement of specific lncRNAs we will focus on genes that have been implicated in DN by evidence from several publications.

The plasmacytoma variant translocation gene *PVT1* had been linked to diabetic nephropathy by the finding that variants in this gene are associated with the development of end-stage renal disease (ESRD) in both type 1 and 2 diabetes mellitus [20,21]. Soon after, it was noted that PVT1 was a non-coding RNA the expression of which was induced by high glucose in mesangial cells. Knockdown of PVT1 significantly decreased the upregulation of both ECM proteins and their transcriptional regulators PAI-1 and TGFβ1 [22], providing a functional link between the genomic data and the pathogenesis of DN. As described above, PVT1 hosts several miRNAs, one of which—miR-1207-5p—could be shown to regulate ECM formation in parallel to the lncRNA itself [17]. Zhang et al. provided further evidence on the role of PVT1 with a component from traditional Chinese medicine (Danggui Buxue Tang) alleviating glucose-indcued proliferation and ECM formation in mesangial cells through targeting PVT1 [23].

MALAT1 is another important example of an lncRNA involved in DN. MALAT1 is induced in the streptozocin-induced diabetic nephropathy mouse model [24]. Based on this finding and further work using cultured podocytes, Hu and colleagues hypothesized MALAT1 to play a role in high-glucose associated podocyte damage involving a feedback loop with beta-catenin employing the MALAT1-binding protein SRSF1 [24]. Furthermore, MALAT1 has been implicated in the damage of other renal cell types in DN. Regarding glomerular endothelial cells MALAT1 induction was accompanied by an epigenetically mediated decrease of Klotho expression [25]. The upregulation of this lncRNA upon glucose exposure led to increased IL1 and TNF-α levels suggesting this lncRNA to be involved in inflammatory processes of the endothelium [26]. In renal tubular epithelial cells MALAT1 induction by high glucose leads to increased pyroptosis by targeting miR-23c and consecutive upregulation of ELAVL1 and NLRP3 [16].

Mitochondrial dysfunction is one of the hallmarks in DN [27]. The modulation of mitochondrial metabolism was linked to the lncRNA TUG1 by an important study in 2016 [28]. TUG1 was differentially expressed in a murine DN model (db/db mice). Its podocyte-specific overexpression in this mouse model improved the glomerular phenotype both regarding albuminuria and histological changes. Mechanistically, TUG1 was linked to mitochondrial bioenergetics by showing that this lncRNA recruits PGC-1α to its own promoter [28]. Further studies corroborate the link of TUG1 to diabetic glomerulopathy. TUG1 both alleviated ECM deposition by acting as a sponge for miR-377 [14] and protected from podocyte apoptosis by modulating ER stress signaling, PGC-1α and TRAF5 [29,30] in different models of DN.

Besides, several lines of evidence point towards a functionally important involvement of the lncRNA NEAT1 in diabetic nephropathy. NEAT1 is induced in a streptozocin-mediated diabetic rat model and murine mesangial cells treated with high-glucose [15,31]. Increased expression of NEAT1 led to the activation of AKT/mTOR signalling accompanied by increased cellular proliferation and fibrosis [31]. Interestingly, this phenotype could be alleviated by knockdown of NEAT1, providing a therapeutic prospective. Additionally, NEAT1 served as a sponge for miR-27b-3p relieving ZEB1—a zinc finger transcription factor associated with epithelial-mesenchymal transition and ECM deposition—from miRNA-mediated repression [15].

CYP4B1-PS1-001 was first described in diabetic nephropathy in a microarray based-screen for dysregulated lncRNAs in the db/db mouse model [32]. Whilst this intergenic lncRNA was strongly downregulated in early phases of DN, its overexpression alleviated the increased proliferative tone in mesangial cells. This effect of CYP4B1-PS1-001 could later on be shown to be mediated by the proteasomal degradation of Nucleolin—a nucleolar ribosome biogenesis factor [33].

More than 20 additional lncRNAs have been implicated in diabetic nephropathy by single publications. Here, we summarize the most important findings. Several of these lncRNAs interact directly with miRNAs and inhibit their function. As examples, LINC01619 induces oxidative podocyte damage by serving as a sponge for miR-27a [34], Gm6135 protects from increased proliferation and apoptosis through impairing the miR-203-3p mediated downregulation of Toll-like receptor 4 [35], lincRNA1700020I14Rik reduces cellular proliferation via inhibition of miR-34-a-5p [36] and lncRNA 150Rik promotes proliferation by sponging miR-451 [37]. Apart from the inhibitory direct binding as described for these examples, lncRNA H19 induces the expression of miR-675 modulating vitamin D receptor expression [38] and lncRNA Erbb4-IR suppresses miR-29b on the transcriptional level [39]. The latter publication is a very good examples of a study taking lncRNAs in diabetic nephropathy beyond a mere description of expression changes by elucidating both the factor driving its expression (Smad3) as well as its downstream effects through mir-29b and by proving the therapeutic potential of Erbb4-IR inhibition in the db/db mouse model. Another lncRNA regulated by Smad3—LRNA9884—promotes DN through the stimulation of inflammation [40]. Similarly, lincRNA Gm4419 promotes inflammation in DN through the NLRP3 inflammasome [41]. Another layer of regulation through lncRNAs that has been found in other systems is epigenetic modifications. As to DN, lncRNA ZEB1-AS1 has been shown to enhance the expression of ZEB1 by promoting H3K4me3 histone modification on its promoter during high glucose treatment with increased ZEB1 exerting an anti-fibrotic role [42]. Importantly, since all of these data were primarily obtained in mouse models, a number of expression screens in DN have been able to show evolutionary conservation of lncRNA modulation in human datasets [43,44,45] indicating the future potential of data obtained in mouse models for the patient setting.

### 2.3. LncRNAs in Other Glomerular Diseases

In comparison to DN, little is known about the contribution of lncRNAs to other glomerular disease entitites. A Pubmed search for lncRNAs AND glomerulus revealed more than 80% of these publications to deal with DN. Fewer than 10 studies report results regarding different types of glomerulonephritis and focal-segmental glomerulosclerosis (FSGS). Due to the scarcity of data, all of these studies are discussed in the following paragraph independent from impact and approach. LncRNA LOC105375913 was found to be increased in tubular cells of 5 FSGS patients [47] and this upregulation was, based on cell culture results, induced by the C3a/p38/XBP-1s pathway. LOC105375913 exerted its profibrotic function through sequestration of miR-27b and consecutive overexpression of Snail. As to actual glomerular changes in FSGS lncRNA LOC105374325 has been described to be upregulated in podocytes of FSGS patients and to induce podocyte apoptosis. Again, the induction of this lncRNA (through p38 and C/EPBbeta) exerts its effects by serving as a sponge for two miRNAs (miR-34c, miR-196a/b) that normally regulate the expression of pro-apoptotic proteins [46]. Interestingly, the profibrotic function of LOC105375913 in the tubulointerstitium and the proapoptotic effect of LOC105374325 in podocytes could also be recapitulated in mouse models overexpressing the respective lncRNAs [46,47]. Regarding direct podocyte injury Fang et al. found the lncRNA GAS5 to be downregulated in a murine sepsis model (using lipopolysaccharide (LPS) injection) [50]. Loss of GAS5 led to a reduction of nephrin and an induction of both Snail/phosphorylated Snail and PI3K/AKT/GSK3β as potential harmful agents. In a descriptive approach Qin et al. reported 10 lncRNAs to be differentially expressed in glomeruli after treatment of mice with Adriamycin based on RNA-sequencing [51] and Gao at el. provided a similar analysis in whole kidney microarray analyses of two glomerulonephritis rat models (nephrotoxic serum nephritis and anti-glomerular basement membrane glomerulonephritis) [52]. As to human samples, two studies described the differential expression of numerous lncRNAs in either IgA-positive or -negative mesangio-proliferative glomerulonephritis [53,54]. The actual pathophysiological impact of this work remains to be determined. One of the longest-known lncRNAs—XIST, which is known for its role in the inactivation of the X-chromosome—has been linked to membranous nephropathy (MN) [48,55]. This lncRNA was found to be upregulated both in a mouse model of MN and in human samples [55]. Data in cell culture pointed towards XIST exerting its proapoptotic effect on podocytes through sequestration of miR-217 and consecutive upregulation of Toll-like receptor 4 [48]. Finally, in a recent report Liao et al. showed differential expression of lncRNAs in kidney biopsies of patients diagnosed with lupus nephritis (LN) [49]. LncRNA RP11-2B6.2 was found upregulated in kidney tissue of LN patients compared to healthy controls. Using HELA and HK-2 cell lines the authors showed that overexpression of RP11-2B6.2 led to inhibition of SOCS1, a known regulator of the IFN-1 signaling pathway and consequently increased the activity of the IFN-1 signaling pathway. They went on to evaluate chromatin accessibility of the SOCS1 locus in the presence and absence of RP11-2B6.2 and determined that downregulation of RP11-2B6.2 coincided with an open chromatin state in the promoter region of SOCS1. Based on this finding Liao et al. proposed that the inhibition of SOCS1 is conveyed by an undetermined epigenetic mechanism [49].

## 3. Tubulointerstitial Disease

**Figure 2:**
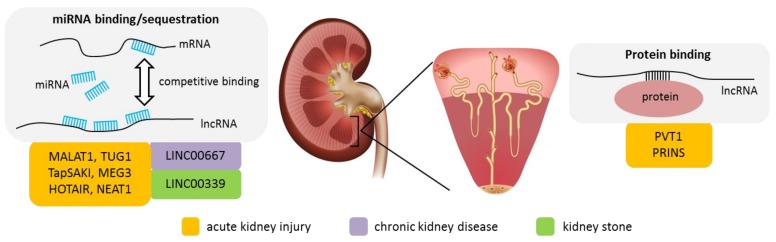
LncRNAs in tubulointerstitial disease and their reported molecular functions. Grey colored boxes depict the molecular mechanisms of lncRNA function (miRNA binding/sequestration and protein binding) reported to play a role in tubulointerstital disease. Orange, purple and green colored boxes indicate the disease: acute kidney injury, chronic kidney disease and kidney stone, respectively. The lncRNAs associated with the particular mechanism and disease are specified by name. Kidney image obtained from: Tefi/shutterstock.com.

To date, the majority of lncRNA studies on tubulointerstitial kidney disease were performed in the context of acute kidney injury (AKI) (Figure 2). Research on chronic kidney disease including genetic disorders such as autosomal dominant polycystic kidney disease (ADPKD) is still very scarce (Table 2).

### 3.1. LncRNAs and Acute Kidney Injury

AKI is a central problem in nephrology considering the high and increasing incidence associated with the demographic changes in Western societies and the lack of specific preventive and therapeutic strategies [56]. Consequently, it is crucial to gain an optimal understanding on the molecular mechanisms predisposing for and protecting from AKI and lncRNAs are an important novel layer of regulation in this context. Major triggers of AKI are sepsis, ischemia reperfusion injury and nephrotoxic agents. In recent years, based on transcriptomic data created from patient material as well as various animal models, a growing number of differentially expressed lncRNAs associated with AKI have been described. The majority of in vivo studies performed to examine AKI were carried out in mice or rats by the induction of ischemia reperfusion injury (IRI) [57,58,59,60,61,62], inflammation stimulated by lipopolysaccharide (LPS) [63,64], urine-derived sepsis [65,66] or exposure to hypoxia [67]. For in vitro models of AKI, the research community mainly relied on the HK-2 human tubular epithelial cell line either treated with LPS [64,65,66,68,69] or grown under hypoxic conditions [59,60,61,62,70]. However, when interpreting these data, it is important to note, that the comparability to the actual patient setting is limited especially for cell culture models. This highlights the importance of a confirmation of the findings in both rodents and especially using human biosamples.

Most evidence for an lncRNA to play a role in AKI has been presented for metastasis-associated lung adenocarcinoma transcript 1 (MALAT1). Initially identified as the most highly induced lncRNA gene in kidney and testis of hypoxic mice, MALAT1 was proposed to be hypoxia-inducible factor (HIF)-2 activated and postulated to function in renal proximal tubuli [67]. MALAT1 expression was suggested to inhibit the hypoxia-induced inflammatory response through the NF-κB pathway [57]. This idea was supported by the findings of Ding et al. who, in an LPS-induced model of AKI, found that MALAT1 interacts with mir-146a—a known regulator of the NF-κB signaling pathway [64]. In spite of these findings, no disease-specific phenotype was observed after challenging MALAT1 knockout mice with IRI-induced AKI [62]. Although the authors suggested that in vivo the impact of MALAT1 on signaling pathways might be minimal, it remains a potential biomarker of kidney IRI due to its high abundance in patient plasma and kidney biopsies [62].

Nuclear enriched abundant transcript 1 (NEAT1) was detected as significantly upregulated in the serum of patients with sepsis-induced as well as in patients with ischemia-induced AKI [71,72]. Using in vitro interaction assays both groups showed that NEAT1 interacts with several miRNAs. In the first case Chen et al. concluded that by binding to miR-204 NEAT1 reduces the cellular level of this miRNA, thereby alleviating the suppression of IL-6R and activating the NF-κB pathway with consecutive inflammation [71]. The second study conducted by Jiang et al. determined miR-27a-3p as a direct interactor of NEAT1 by RNA immunoprecipitation [72]. In knockdown and overexpression experiments the authors showed the negatively correlated influence of NEAT1 on miR-27a-3p levels leading to apoptosis.

Similar studies link the lncRNAs HOX transcript antisense RNA (HOTAIR) [66], taurine upregulated gene 1 (*TUG1*) [73], maternally expressed gene 3 (*MEG3*) [63] and transcript predicting survival in AKI (*TapSAKI*) [65] to a range of other miRNAs.

HOTAIR—known to play an important role in apoptosis—was upregulated in rats with sepsis caused by urinary tract infection [66]. The authors further observed a negative regulation of miR-22 and induction of apoptosis in HK-2 cells and related this regulation to the stabilization of high-mobility-group-protein B1 (HMGB1)—a key mediator of inflammation and a known target of miR-22. In a study using HOTAIR mimics in vivo in rats with sepsis induced AKI, Jiang et al. found that expression of HOTAIR declined serum serine—as well as blood urea nitrogen levels and reduced signs of apoptosis in kidney tissue [74]. These effects were attributed to the concomitant reduction of miR-34a and increase of B-cell leukemia/lymphoma 2 (Bcl-2) protein levels, with Bcl-2 being an anti-apoptotic factor and a target of miR-34a.

To unravel the role of the lncRNA TUG1, detected at lower levels in the serum of patients suffering from sepsis-associated AKI in comparison to healthy controls, a LPS based in vitro model was used [73]. Despite the fact that this study examined rat mesangial cells we report their results in this section due to the link to other LPS-induced AKI models. Liu et al. found that overexpression of TUG1 reversed the deteriorating effects of LPS on RMCs and showed that this was mediated by miR-142-3p. Furthermore, SIRT1 a known suppressor of the NF-κB signaling pathway, was identified as the direct target downregulated by miR-142-3p in the absence of TUG1. Consequently, these findings suggest that the lower levels of TUG1 observed in the serum of AKI patients may lead to activation of the NF-κB pathway driving inflammation. These findings are supported by a study of Xu et al. confirming the protective effect of overexpressed TUG1 on LPS-induced injury in HK-2 cells [68]. Although, the conveying factor between TUG1 and SIRT1 was found to be miR-223 in this study, the effects on the NF-κB pathway were similar. In addition, the authors suggested that the protective effects of TUG1 on renal tubular epithelial cells injury are associated with activation of the PI3K/AKT pathway.

In two individual studies MEG3 was found to be upregulated after LPS or hypoxia treatment, respectively and in both cases the authors report the sequestration of miRNAs by MEG3 [58,63]. Yang et al. showed that the higher levels of MEG3 lead to increased binding of miR-21 removing it from the pool available to inhibit the translation of programmed cell death protein 4 (PDCD4) [63]. The downregulation of MEG3 resulted in the inhibition of PDCD4 and attenuation of LPS-induced apoptosis. Likewise, data presented by Pang et al. suggested MEG3 to sequester mir-181b leading to upregulation of TNF-α in hypoxia-induced kidney injury in acute renal allografts [58].

TapSAKI, initially discovered as a circulating lncRNA upregulated in the plasma of AKI patients and their kidney tissue was proposed as a biomarker with predictive value for the survival of AKI patients [75]. Recently, Shen et al. using a urine-derived sepsis model of AKI in rats and LPS treated HK-2 cells, showed that TapSAKI interacts with miR-22 [65]. In a state of TapSAKI upregulation this interaction leads to increased levels of phosphatase and tensin homolog (PTEN) and activation of TLR4 and NF-κB pathway triggering inflammation.

In the section below we discuss lncRNAs which so far have only a single study connecting them to acute kidney injury—plasmacytoma variant translocation 1 (PVT1) [69], psoriasis-susceptibility-related RNA gene induced by stress (PRINS) [59], growth arrest-specific 5 (GAS5) [61], aspartyl-tRNA synthetase anti-sense 1 (DARS-AS1) [70], LINC00520 [60], UC.173 [76].

An involvement of PVT1 in kidney disease has been described previously for diabetic nephropathy [22] and is discussed above in the DN section. In their 2017 study on LPS-treated HK-2 cells, Huang et al. found PVT1 significantly upregulated compared to untreated control cells [69]. The authors reported PVT1 overexpression to decrease cell viability and to trigger inflammatory responses. Vice versa, downregulation of PVT1 resulted in suppression of inflammatory factors by regulation of the JNK/NF-κB signaling pathways. The authors suggested the binding of TNF-α by PVT1 to be the responsible mechanism leading to inhibition of JNK/NF-κB signaling pathways promoting inflammatory responses in sepsis-induced AKI.

The expression and secretion of RANTES/CCL5 (regulated on activation, normal T cell expressed and secreted) is known to recruit circulating leukocytes to sites of injury and to reinforce inflammatory reactions. In their report, Yu et al. showed that PRINS, a HIF-1α regulated lncRNA is potentially involved in RANTES production [59]. The study suggested a significant upregulation and direct interaction between PRINS and RANTES in hypoxic conditions leading to enhanced inflammation and AKI progression.

The hypoxia-responsive lncRNA GAS5 was reported to be upregulated in IRI treated mice by Geng et al. [61]. The increase of GAS5 was linked to induction of proapoptotic factors: p53, cIAP2 and TSP-1. Performing in vitro experiments in hypoxia treated HK-2 cells the authors confirmed this correlation for p53 and TSP-1 and in the reciprocal approach showed that knockdown of GAS5 led to downregulation of p53 and TSP-1, attenuating apoptosis.

The study conducted on HK-2 and primary renal proximal tubular epithelial cells (RPTEC) by Mimura et al. compared lncRNA expression patterns between hypoxic and normoxic cells [70]. DARS-AS1 containing hypoxia-responsive elements (HRE) in the promoter region was found upregulated as a consequence of HIF-1α binding in hypoxic conditions. The authors further showed that the expression of DARS-AS1 has inhibitory effects on cell death progression and therefore may be important for the survival of renal tubular cells during AKI.

Tian et al. observed an upregulation of LINC00520 in rat kidney tissue following IRI treatment [60]. Using HK-2 cells they determined a relation between LINC00520 and miR-27b and suggested that LINC00520 may inhibit miR-27b by competitive binding. Reduction of miR-27b in the cellular pool resulted in the upregulation of Oncostatin M receptor (OSMR). Finally, the authors showed knockdown of LINC00520 to reduce levels of OSMR, reduction of PI3K/AKT and attenuation of renal injury in rats.

UC.173 belongs to a group of lncRNAs transcribed from an ultra-conserved region (T-UCR) shared between human, rat and mouse genomes and was initially reported as downregulated in lead-exposed human populations and animal models [77]. Qin et al. studied UC.173 in the context of lead-induced renal tubular epithelial cell apoptosis and showed that lead exposure reduced the levels of this lncRNA in HK-2 and HKC cells [76]. While the overexpression of UC.173 had no effect on cell viability, cell cycle or apoptotic factors in lead-unexposed HK-2 and HKC cells, its overexpression in lead treated cells increased cell survival and showed reduced signs of apoptosis.

### 3.2. LncRNAs Associated with Other Tubulointerstitial Diseases

In this section we give an overview of recent advances with regard to the function of lncRNAs in tubulointerstitial diseases other than acute kidney injury. We discuss the role of lncRNAs in 5/6 nephrectomy, autosomal dominant polycystic kidney disease (ADPKD) and kidney injury by crystal-formation (e.g., calcium oxalate).

To date, two lncRNAs have been associated with the transcriptional response in rat models of 5/6 nephrectomy [78,79]. LINC00963, studied by Chen et al. due to its upregulation in the context of renal interstitial fibrosis and oxidative stress, was determined to influence FoxO3a levels by an undetermined mechanism [79]. Lowered LINC00963 levels were associated with the activation of the FoxO signaling pathway, consequently leading to suppression of renal interstitial fibrosis and oxidative stress. The same group published a study investigating the role of LINC00667 [78]. LINC00667 was upregulated while miR-19b-3p was downregulated in kidney tissue of patients suffering from CKD (chronic kidney disease) of heterogenous etiology compared to normal renal tissue. The authors linked the downregulation of miR-19b-3b to a sequestration by LINC00667. In the 5/6 nephrectomy rat model used in this study, overexpression of miR-19b-3p decreased levels of TGF-β1, CTGF, α-SMA and TIMP-1 and improved the renal damage. A recent review provides a detailed view on the general role of non-coding RNAs in renal fibrosis [84].

To the best of our knowledge, so far only one lncRNA has been shown to have functional implications in ADPKD. Aboudehen et al. investigated the role of Hoxb3os, an lncRNA abundantly expressed in the kidney and evolutionary conserved with the human ortholog HOXB-AS1 [80]. The expression of HOXB-AS1 was decreased in the kidney tissue of ADPKD patients and likewise in *Pkd1* and *Pkd2* knockout mice, a genetic model of ADPKD, a downregulation of the mouse ortholog was observed. In a cell model using mIMCD3, the authors showed that knockout of Hoxb3os leads to mTORC1 activation and increase in mitochondrial respiration and that re-expression of Hoxb3os rescues this phenotype.

Recent findings have also shown that lncRNAs are associated with renal injury mediated by crystal formation. The studies - mainly based on HK-2 cell exposure to calcium oxalate monohydrate (COM), the major constituent of kidney stones [85], range from profiling changes in lncRNA expression upon COM stimulation to analysis of individual lncRNAs. Wang et al.—using the same cell model—profiled transcriptional changes by RNA sequencing and determined 25 differentially expressed lncRNAs [86]. Cao et al. used an ethylene glycol-induced rat model of kidney stone formation to profile expression changes and found 1440 lncRNAs differentially regulated in comparison to untreated controls [87]. In addition to an in vitro COM-HK-2 cell model, Zhang et al. used a mouse model of calcium oxalate-induced kidney damage and found 376 differentially regulated lncRNA in COM-treated animal in comparison to the control group. 15 of the regulated lncRNAs had homologous genes in the human genome. LncRNA AU015836 and the human homolog CHCHD4P4 were upregulated upon COM treatment in mouse kidney tissue and HK-2 cells, respectively. The authors further showed that CHCHD4P4 was involved in epithelial–mesenchymal transition (EMT) by regulation of EMT-related genes and the inhibited cell proliferation in COM-exposed HK-2 cells [82]. In their study on LINC00339 in COM-treated HK-2 cells, Song et al. linked the promotion of renal tubular epithelial pyroptosis to the activation of the NLRP3 inflammasome [81]. The inflammasome was activated due to increased levels of NLRP3 resulting from the sequestration of miR-22-3p—a regulator of NLRP3—by LINC00339. As a last example, antisense non-coding RNA in the INK4 locus ANRIL, a highly expressed lncRNA in the serum of patients suffering from uric acid nephropathy (UAN), was studied by Hu et al. in HK-2 cells and a rat model of UAN [83]. The authors report that downregulation of ANRIL in the animal model resulted in reduced signs of renal injury. According to the results from the in vitro studies in HK-2 cells this improvement was conveyed through the reduced sequestering of miR-122-5p by ANRIL, which in turn led to the downregulation of the BRCA1-BRCA2-containing complex subunit 3 (BRCC3) and as a consequence suppressed the activation of NLRP3 inflammasome.

## 4. Systemic Kidney Biomarkers

Regarding glomerular disease there are no reports on circulating lncRNAs as potential biomarkers. For membranous nephropathy lncRNA XIST has been hypothesized to be a potential urinary biomarker [55]. However, the increase of urinary XIST—which was originally described in kidneys of a mouse model of MN—was not specific to MN but rather reflected injury in different types of glomerulonephritis. Besides, the lncRNA TapSAKI was identified as a circulating factor with the potential to predict mortality among a cohort of 109 patients suffering from acute kidney injury [75]. A potential implementation of lncRNAs as biomarkers for standardized clinical use will require studies in larger cohorts including questions on specificity and predictive value regarding clinical outcome. Since for most renal disorders little is known regarding the impact of lncRNAs, screening studies describing differential expression of lncRNAs using state-of-the-art methodology (e.g., RNA and single cell RNA sequencing) are still an important asset but will require further complementation by targeted analyses elucidating the molecular function of specific lncRNAs (e.g., RNAscope for visualization, quantitative polymerase chain reaction (qPCR) for quantification and CHART-MS for the identification of binding partners.

## 5. Conclusion and Outlook

LncRNAs provide a fascinating new layer to pathophysiological studies and the search for novel therapeutic strategies in kidney disease. However, apart from few examples such as diabetic nephropathy and acute kidney injury, little is known about their impact in renal pathologies and most studies have remained primarily descriptive. Future work will need to close this gap in order to increase our understanding of this new class of non-coding RNAs in nephrology and to eventually elucidate how we can exploit the potential of lncRNA modulation for patients suffering from kidney disease. The inhibition of lncRNAs in vivo is technically no more challenging than the inhibition of messenger RNAs; therefore, with an increasing number of antisense oligonucleotide therapies coming into clinical use at the moment (e.g., RNAi for TTR-amyloidosis or modulation of splicing for spinal muscular atrophy) this concept could be of high interest for targeting lncRNAs in kidney disease [88,89,90]. Regarding the cell-type specific expression of RNAs in the kidney and specifically the glomerulus single cell or single nucleus RNA sequencing (scRNAseq) studies have started to add much to our knowledge in both conditions of health and disease including DN [91,92,93,94]. Importantly, since nearly all lncRNAs are polyadenylated, the commonly used 3′end sequencing approaches in scRNAseq do capture these sequences as well if samples are sequenced at a sufficient depth (due to comparably lower transcript counts of lncRNAs compared to other RNA species). These approaches will now need to be analyzed in detail regarding lncRNAs and transferred to human kidney biopsies using either scRNAseq itself (for an example regarding a first approach in Lupus nephritis see [95]) or targeted imaging of specific lncRNAs, e.g., by single-molecule FISH. When using model organisms, the difficulties in predicting evolutionary conservation are still a major challenge, which will make innovative bioinformatics solutions an essential asset. Furthermore, the function of lncRNAs can hardly be predicted and its elucidation requires continuous development of novel techniques. Taken together, it is time for nephrologists to team up with molecular and computational biologists and unravel the full impact of lncRNAs in kidney disease.

## Figures and Tables

**Table 1:** LncRNAs involved in glomerular diseases.

Diabetic Nephropathy				
lncRNA	main disease model	suggested function	target	reference
TUG1	diabetic mice (db/db)	transcriptional activation	Ppargc1a promoter	[28]
	diabetic mice (db/db)	miRNA binding	miR-377	[14]
	streptozotocin treated rats	protein binding	TRAF5	[29]
MALAT1	streptozotocin treated rats	miRNA binding	miR-23	[16]
	streptozotocin treated mice	expression changed	IL-6 and TNF-α	[26]
	streptozotocin treated mice	expression changed	β-catenin, SRSF1	[24]
NEAT1	streptozotocin treated rats	miRNA binding	miR-27b-3p	[15]
	streptozotocin treated rats	expression changed	Akt/mTOR signaling	[31]
PVT1	high glucose treated CIHP-1	expression changed	ECM-related proteins	[22]
ZEB1-AS	streptozotocin treated mice	recruitment of histone modifications	ZEB1 promoter	[42]
LRNA9884	diabetic mice (db/db)	transcriptional activation	MCP-1	[40]
LINC01619	streptozotocin treated rats	miRNA binding	miR-27a	[34]
Gm6135	diabetic mice (db/db)	miRNA binding	miR-203-3p	[35]
CYP4B1-PS1-001	diabetic mice (db/db)	protein binding	NCL	[33]
1700020I14Rik	diabetic mice (db/db)	miRNA binding	miR-34	[36]
150Rik	diabetic mice (db/db)	miRNA binding	miR-451	[37]
H19	vitamin D3 treated CIHP-1	expression changed	miR-675	[38]
Erbb4-IR	diabetic mice (db/db)	transcriptional repression	miR-29b	[39]
Gm4419	high glucose treated mouse MCs	protein binding	p50	[41]
Focal-Segmental Glomerulosclerosis				
lncRNA	main disease model	suggested function	target	reference
LOC105374325	adriamycin treated podocytes	miRNA binding	miR-34c and miR-196a/b	[46]
LOC105375913	FSGS patient serum treated HK-2	miRNA binding	miR-27b	[47]
Membranous Nephropathy				
lncRNA	main disease model	suggested function	target	reference
XIST	angiotensin II treated AB8/13	miRNA binding	miR-217	[48]
Lupus Nephritis				
lncRNA	main disease model	suggested function	target	reference
RP11-2B6.2	IFN-I treated HeLa and HK-2	epigenetic inhibition	SOCS1	[49]

**Table 2:** LncRNAs involved in tubulointerstitial disease.

Acute Kidney Injury				
lncRNA	main disease model	suggested function	target	reference
MEG3	IRI in renal allografts	miRNA binding	miR181b-5p	[58]
	LPS treated mice	miRNA binding	miR-21	[63]
NEAT1	LPS treated RMCs	miRNA binding	miR-204	[71]
	CoCl2 treated HK-2	miRNA binding	miR-27a-3p	[72]
MALAT1	LPS treated rats	miRNA binding	miR-146a	[64]
	hypoxia treated mice	expression changed	unknown	[67]
TapSAKI	Urine derived sepsis in rats	miRNA binding	miR-22	[65]
	AKI patients	circulating biomarker	unknown	[75]
HOTAIR	Urine derived sepsis in rats	miRNA binding	miR-22	[66]
	CLP induced sepsis in rats	expression changed	miR-34a and Bcl-2	[74]
TUG1	LPS treated RMCs	miRNA binding	miR-142-3p	[73]
	LPS treated HK-2	miRNA binding	miR-223	[68]
LINC00520	IRI in rats	miRNA binding	miR-27b-3p	[60]
PVT1	LPS treated HK-2	protein binding	TNF-α	[69]
PRINS	IRI in mice	protein binding	RANTES (CCL-5)	[59]
GAS5	IRI in mice	expression changed	mRNA of p53 and TCP1	[61]
DARS-AS1	hypoxia treated HK-2 and RPTECs	expression changed	unknown	[70]
UC.173	lead treated HK-2 and HKC	expression changed	unknown	[76]
Chronic kidney disease				
lncRNA	main disease model	suggested function	target	reference
LINC00667	CKD patients and rat model	miRNA binding	miR-19b-3p	[78]
LINC00963	CKD rat model	expression changed	mRNA of FoxO3a	[79]
Autosomal Dominant Polycystic Kidney Disease				
lncRNA	main disease model	suggested function	target	reference
Hoxb3os	Pkd1/2 knockout mice	expression changed	unknown	[80]
Kidney stone				
lncRNA	main disease model	suggested function	target	reference
LINC00339	COM treated HK-2	miRNA binding	miR-22-3p	[81]
CHCHD4P4	COM treated mice	expression changed	unknown	[82]
Uric Acid Nephropathy				
lncRNA	main disease model	suggested function	target	reference
ANRIL	uric acid treated HK-2	miRNA binding	miR-122-5p	[83]

## Abbreviations

CIHP-1	human podocyte cell line
MC	mesangial cells
FSGS	focal-segmental glomerulosclerosis
HK-2	human proximal tubule epithelial cell line
AB8/13	human podocyte cell line
IRI	ischemia reperfusion injury
LPS	lipopolysaccharide
RMCs	rat mesangial cell line
HK-2	human proximal tubule epithelial cell line
CLP	cecal ligation puncture
RPTECs	human renal proximal tubular epithelial cell line
HKC	human kidney proximal tubular epithelial cell line
CKD	chronic kidney disease
COM	calcium oxalate monohydrate
